# Isolation and Characterization of Microsatellite Loci for the Isopod Crustacean *Armadillidium vulgare* and Transferability in Terrestrial Isopods

**DOI:** 10.1371/journal.pone.0076639

**Published:** 2013-10-03

**Authors:** Isabelle Giraud, Victorien Valette, Nicolas Bech, Frédéric Grandjean, Richard Cordaux

**Affiliations:** Université de Poitiers, UMR CNRS 7267 Ecologie et Biologie des Interactions, Equipe Ecologie Evolution Symbiose, Poitiers, France; Louisiana State University, United States of America

## Abstract

*Armadillidium vulgare* is a terrestrial isopod (Crustacea, Oniscidea) which harbors *Wolbachia* bacterial endosymbionts. *A. vulgare* is the major model for the study of *Wolbachia*-mediated feminization of genetic males in crustaceans. As a consequence of their impact on host sex determination mechanisms, *Wolbachia* endosymbionts are thought to significantly influence *A. vulgare* evolution on various grounds, including population genetic structure, diversity and reproduction strategies. To provide molecular tools for examining these questions, we isolated microsatellite loci through 454 pyrosequencing of a repeat-enriched *A. vulgare* genomic library. We selected 14 markers and developed three polymorphic microsatellite multiplex kits. We tested the kits on two *A. vulgare* natural populations and found high genetic variation, thereby making it possible to investigate the impact of *Wolbachia* endosymbionts on *A. vulgare* nuclear variation at unprecedented resolution. In addition, we tested the transferability of these kits by cross-species amplification in five other terrestrial isopod species harboring *Wolbachia* endosymbionts. The microsatellite loci showed good transferability in particular in *Armadillidium nasatum* and *Chaetophiloscia elongata*, for which these markers represent promising tools for future genetic studies.

## Introduction

*Armadillidium vulgare* is a terrestrial isopod (Crustacea, Oniscidea) which exhibits a worldwide distribution. *A. vulgare* harbors alpha-proteobacterial endosymbionts of the genus *Wolbachia* [1], [2]. These maternally-inherited, intracytoplasmic bacteria are known to manipulate host reproduction to enhance their own transmission through four different mechanisms: cytoplasmic incompatibility, thelytokous parthenogenesis, male killing and feminization [3], [4]. *Wolbachia* endosymbionts are prevalent in terrestrial isopods [5], and *A. vulgare* has emerged as a major model for studying *Wolbachia*-mediated feminization [3], [6]–[8]. In *A. vulgare*, zygotes carrying *Wolbachia* develop a female phenotype, whatever their sex chromosome composition. In particular, genetic males harboring *Wolbachia* are converted into functional females. As a consequence, *A. vulgare* populations in which *Wolbachia* are present show sex ratio distortions towards females, thereby enhancing *Wolbachia* spread in infected populations. In addition, some *A. vulgare* individuals carry another feminizing factor, known as the f element, which may be a fragment of the *Wolbachia* genome carrying feminization information and transferred into the host nuclear genome [3], [9]. Furthermore, the occurrence of multiple feminizing factors has generated genetic conflicts in this system, which resulted in the selection of *A. vulgare* nuclear genes resisting feminization [3], [10], [11]. Thus, sex determination mechanisms are very dynamic in *A. vulgare*, outlining the prime influence of *Wolbachia*. These endosymbionts are thought to impact *A. vulgare* evolution on various additional grounds, including population genetic structure, diversity and reproduction strategies [12].

Mitochondrial DNA markers have been used in several studies, indicating relatively high variability in *A. vulgare* [1], [13]–[15] as compared to other terrestrial isopod species such as *Porcellionides pruinosus* [16]. A more elaborate understanding of *A. vulgare*/*Wolbachia* interactions would benefit from information on *A. vulgare* nuclear variation. Recently, five microsatellite markers [17] were used to investigate nuclear variation in *A. vulgare* populations from western France, suggesting a genetic structure compatible with isolation by distance [14]. Although polymorphic, these markers may not be in sufficient number to offer the desired resolution for detecting a possibly subtle impact of *Wolbachia* on *A. vulgare* nuclear variation, population dynamics and evolution. To provide tools for examining these questions, we isolated microsatellite loci through 454 pyrosequencing of a repeat-enriched *A. vulgare* genomic library. We selected markers yielding clear amplification signals and showing appropriate polymorphism levels. Next, we used the candidate loci to develop three polymorphic microsatellite multiplex kits. The transferability of these kits was tested by cross-species amplification in other terrestrial isopod species harboring *Wolbachia* endosymbionts [5], [18].

## Materials and Methods

### Ethics Statement

No ethics statement was required for the described study. No specific permission was required for sampling the two *A. vulgare* field populations (La Crèche and Beauvoir-sur-Niort, France) because they were located in public areas. Field populations of *Armadillidium nasatum* (Poitiers, France) and *P. pruinosus* (Buxerolles, France) were sampled on private lands after the land owners gave permission to conduct sampling on the sites. None of these species is an endangered or protected species.

### Microsatellite isolation

For genomic library construction, we maximized genomic diversity by using eight *A. vulgare* female individuals selected from the following laboratory lines: BF (bac 377), BH (bac 366), CY (bac 291), CW (bac 49), POA (bac 42), WS (bac 45), WX (matricule 1288) and ZM (bac 47). Total genomic DNA was obtained for each individual by standard phenol-chloroform extraction [19] followed by RNase (10 mg/ml) treatment. DNA concentration was measured using a picogreen assay and equimolar amounts of the eight samples were pooled. The pooled sample was used by GenoScreen (Lille, France) to construct a microsatellite-enriched genomic library, as previously described [20]. The library was sequenced by GenoScreen in a partial 454 GS FLX sequencer run with Titanium chemistry, as previously described [20]. The resulting reads were analyzed with the QDD software [21] to identify reads containing microsatellite motifs and design primers for PCR amplification.

### Locus validation and polymorphism tests

All microsatellite loci with PCR primers designed using QDD were initially tested using two *A. vulgare* female individuals from our laboratory line BF (matricule 2756).Total genomic DNA from the two samples was extracted as above and subjected to whole genome amplification using the illustra GenomiPhi V2 DNA Amplification Kit (GE Healthcare) to generate large enough amounts of template DNA for microsatellite testing. To reduce genotyping costs, each locus was amplified and fluorescently labeled using the M13(-21) primer genotyping protocol [22]. This PCR method uses three primers: a locus-specific forward primer with M13(-21) tail at its 5′ end, a locus-specific reverse primer and a universal 6_FAM-labeled M13(-21) primer. PCR amplification was performed in 10 µL reactions, using 0.5 µM of both 6_FAM-M13(-21) and reverse primers, 0.125 µM forward primer, 0.25 U GoTaq DNA Polymerase (Promega), 1X PCR reaction buffer (Promega), 0.2 mM dNTPs (Promega) and 1 µL DNA template. PCR thermal conditions were as previously described [22]. Subsequently, 0.5 µL PCR products were added to 9 µL formamide and 0.35 µL ROX standard (Life Technologies), and resolved by electrophoresis on an ABI PRISM 3130 Genetic Analyzer. Product sizes were determined using the GeneMapper software (Applied Biosystems), followed by eye verification.

Microsatellite loci amplifying in at least one of the two tested individuals and yielding unambiguous amplification signals were further evaluated for their informativeness. Amplification success rates and number of different alleles at each locus were assessed by genotyping a panel of 24 *A. vulgare* individuals (12 males and 12 females) from five laboratory lines: BF (n = 5), BFog (n = 4), WXa (n = 5), BG (n = 5) and ZM (n = 5). First, the 24 samples were subjected to whole genome amplification as described above. Next, microsatellite loci were genotyped using the M13 (-21) primer protocol described above.

### Multiplexing and cross-species amplification

Based on the genotyping results of the 24-individual panel, we selected 14 microsatellite markers for which locus-specific forward primers (without M13(-21) tail) were ordered with labeled dyes (6_FAM, HEX or NED) (Table 1). First, we verified amplification of the 14 markers in simplex PCR conditions on three individuals from each of two *A. vulgare* field populations (La Crèche and Beauvoir-sur-Niort, France, Table 2). Total genomic DNA was extracted as above. All PCR reactions were carried out using the QIAGEN multiplex PCR kit according to the manufacturer's standard microsatellite amplification protocol in a final volume of 10 µL, with an annealing temperature of 58°C and a final concentration of 0.2 µM for each primer. DNA concentrations were adjusted for all individuals between 20 and 60 ng/µL. Next, 1 µL PCR product was added to 18 µL formamide and 0.5 µL ROX standard (Life Technologies). PCR products were resolved by electrophoresis and their size determined as described above.

**Table 1:** Microsatellite multiplex kits developed for the terrestrial isopod *Armadillidium vulgare*.

Locus name	Repeat motif	Forward primer (5′-3′)	Reverse primer (5′-3′)	Dye
Multiplex kit #1				
AV0023	AG	TGGAATTTATGTTTGGAGAGGG	GAGGTTAAGTCTGGGGTCGG	HEX
AV0056	GTT	TTCAAAGGAGCGTTTGACCT	AACCACAGCAACAACAGCAG	6_FAM
AV0085	GTA	CATGCCGTAAGTCCTCTAGACA	TGTGTTATGGTAATTACATTGAAGTTT	NED
AV0086	TTC	CCCTTGGCTTCCGATACTT	TGTCCACAAAGCCAAAATGA	HEX
AV0096	AAC	TGGCATAAACCAGCTATAAACC	TAGTTGCTTTTCCCCTACTTTTG	6_FAM
Multiplex kit #2				
AV0002	ACTCCG	CGACTCCGACTCCGAATG	TTCCGACATGTACGATTTTATCA	6_FAM
AV0016	TC	GCTCATTTATGATCTCGTCGC	CTCCCACGTGGTTGATCTTC	6_FAM
AV0018	CAA	GAAGAAATTCAAACTTCACCATCA	CTTTGAACAGACTTACGAATAACATC	HEX
AV0032	TC	TTTCAACCTTCCTAACCAAACC	TTGTTTTATATCCACGACCATCC	NED
AV0099	TG	CCCCATTTGTGCATGTAGTG	ACCCCTCGCTTACATTACCC	HEX
Multiplex kit #3				
AV0061	CT	GTTTGTATGCATTTACCCCTCTTC	GTATGGAACGAAGGGACCG	HEX
AV0063	TACA	CAAAACATCTGTACGGATTCCC	GCCAAACATAAATGCTCGCT	NED
AV0089	CTA	TTGTTACTTCTACCACCACTATTGC	TGGCTCTATAATGATCAATGGAA	HEX
AV0128	GAT	TGTCGTTGTGAACAGGCTAAA	CGTCCGTCGAATGATATTTGT	6_FAM

**Table 2:** Characterization of the 14 microsatellite loci used in multiplex kits in two *Armadillidium vulgare* populations.

Populations	La Crèche (N = 20)			Beauvoir-Sur-Niort (N = 20)			Overall population (N = 40)			
GPS coordinates	46°21′40.08011″N, 00°18′21.95247″W			46°10′35.91493″N, 00°28′30.45661″W						
Parameters	Na	He/Ho	Fis	Na	He/Ho	Fis	Na	Size range (bp)	He/Ho	Fis
Multiplex kit #1										
AV0023	1	-	-	1	-	-	1	193	-	-
AV0056	4	0.57/0.70	−0.23	5	0.46/0.50	−0.09	6	198–219	0.52/0.60	−0.15
AV0085	2	0.10/0.10	−0.03	4	0.28/0.30	−0.09	4	175–190	0.19/0.20	−0.06
AV0086	3	0.10/0.10	−0.01	3	0.23/0.25	−0.09	3	113–122	0.17/0.18	−0.06
AV0096	2	0.51/1.00	**−1.00**	3	0.55/1.00	**−0.87**	3	83–107	0.52/1.00	**−0.94**
Multiplex kit #2										
AV0002	5	0.51/0.45	0.12	5	0.54/0.61	−0.15	5	260–308	0.52/0.53	−0.02
AV0016	3	0.34/0.30	0.11	2	0.14/0.15	−0.06	3	114–126	0.24/0.23	0.08
AV0018	4	0.71/0.70	0.02	5	0.77/0.85	−0.10	5	97–136	0.74/0.78	−0.04
AV0032	4	0.43/0.45	−0.04	3	0.38/0.35	0.07	4	89–105	0.40/0.40	0.00
AV0099	5	0.49/0.20	**0.60**	6	0.71/0.58	0.19	6	160–198	0.60/0.39	**0.36**
Multiplex kit #3										
AV0061	1	-	-	1	-	-	1	138	-	-
AV0063	3	0.45/0.50	−0.12	3	0.50/0.40	0.20	3	129–137	0.48/0.45	0.05
AV0089	2	0.10/0.10	−0.03	1	-	-	2	85–88	0.05/0.05	−0.01
AV0128	2	0.51/0.95	**−0.90**	3	0.45/0.61	−0.37	3	120–129	0.49/0.79	**−0.61**

Sampled locations, GPS coordinates (longitude and latitude in World Geodetic System 1984) and number of sampled individuals (N) are shown. Number of alleles (Na), size range of alleles (bp), unbiaised expected heterozygosity (He), observed heterozygosity (Ho), and Fis are shown. Significant values (*P*≤0.01) are shown in bold.

After simplex PCR verification, we pooled the 14 markers in three multiplex kits (Table 1) according to amplified fragment sizes and dyes to maximize efficiency and minimize costs. The multiplex kits were tested with the same three individuals used for simplex PCR reactions, using the QIAGEN multiplex PCR kit as described above. Identical results were obtained for both simplex and multiplex PCR conditions, thereby validating the use of the three multiplex kits in subsequent analyses. Polymorphism of the 14 microsatellite loci in *A. vulgare* field populations was evaluated by genotyping 20 individuals from each of two populations (La Crèche and Beauvoir-sur-Niort, France) using the multiplex kits.

To investigate transferability of the 14 microsatellite markers, cross-species amplifications were performed in five terrestrial isopod species related to *A. vulgare* and known to harbor *Wolbachia* endosymbionts [5], [18]: *A. nasatum* (n = 8) and *P. pruinosus* (n = 8), which were sampled in the field in 2012, and *Chaetophiloscia elongata* (n = 8), *Porcellio scaber* (n = 8) and *Oniscus asellus* (n = 8) from laboratory populations (Table 3).Total genomic DNA was extracted as above. Genotyping was performed using the three multiplex kits.

**Table 3:** Transferability of the 14 microsatellite loci used in multiplex kits in five terrestrial isopod species.

Species	*Armadillidium nasatum*				*Porcellionides pruinosus*				*Chaetophiloscia elongata*				*Oniscus asellus*				*Porcellio scaber*			
Populations	Poitiers				Buxerolles				Laboratory line				Laboratory line				Laboratory line			
GPS coordinates	46°35′3.77006″N, 00°22′16.07919″E				46°36′50.3″N, 00°21′38.6″E															
Parameters	Na	Size range	He/Ho	Fis	Na	Size range	He/Ho	Fis	Na	Size range	He/Ho	Fis	Na	Size range	He/Ho	Fis	Na	Size range	He/Ho	Fis
Multiplex kit #1																				
AV0023	IN	-	-	-	IN	-	-	-	1	193	-	-	IN	-	-	-	IN	-	-	-
AV0056	3	198–209	0.24/0.13	0.5	IN	-	-	-	2	209–219	0.13/0.13	-	IN	-	-	-	6	231–279	0.85/0.43	**0.51**
AV0085	4	169–178	0.74/0.38	**0.51**	IN	-	-	-	2	175–181	0.13/0.13	-	IN	-	-	-	1	175	-	-
AV0086	1	110	-	-	3	107–113	0.62/0.29	0.56	2	110–113	0.48/0.67	−0.43	2	110–113	0.53/0.17	0.71	2	110–113	0.13/0.13	-
AV0096	4	77–89	0.70/0.71	−0.02	3	74–89	0.44/0.50	−0.15	IN	-	-	-	2	74–83	0.53/0.50	0.06	3	74–89	0.59/0.17	**0.74**
Multiplex kit #2																				
AV0002	IN	-	-	-	IN	-	-	-	IN	-	-	-	IN	-	-	-	IN	-	-	-
AV0016	2	94–116	0.36/0.14	0.63	IN	-	-	-	2	98–116	0.53/0.83	−0.67	IN	-	-	-	IN	-	-	-
AV0018	3	100–106	0.54/0.38	0.32	IN	-	-	-	3	97–135	0.59/0.67	−0.14	IN	-	-	-	IN	-	-	-
AV0032	3	89–99	0.73/0.60	**0.2**	IN	-	-	-	2	89–99	0.47/0.60	−0.33	IN	-	-	-	3	95–99	0.32/0.33	−0.05
AV0099	3	160–174	0.69/0.63	0.1	IN	-	-	-	1	160	-	-	IN	-	-	-	IN	-	-	-
Multiplex kit #3																				
AV0061	2	144–146	0.50/0.25	0.52	IN	-	-	-	2	138–146	0.13/0.13	-	IN	-	-	-	IN	-	-	-
AV0063	2	133–137	0.20/0.20	-	3	129–137	0.54/0.43	0.22	3	129–137	0.67/0.67	0	IN	-	-	-	IN	-	-	-
AV0089	IN	-	-	-	2	85–88	0.53/0.50	0.06	2	85–88	0.33/0.38	−0.17	2	85–88	0.36/0.40	−0.14	2	85–88	0.17/0.17	-
AV0128	IN	-	-	-	IN	-	-	-	2	126–129	0.53/0.80	−0.6	IN	-	-	-	IN	-	-	-

Parameters are as described in the legend to Table 2. IN indicates inconsistent amplification of the locus in one species.

### Data analyses

To assess genetic variability and transferability of our microsatellite markers, we calculated number of alleles (Na), unbiased expected heterozygosity (He) [23], observed heterozygosity (Ho) and Fis [24] using Genetix version 4.05.2. We computed these genetic indices from two *A. vulgare* populations and from individuals of the five other aforementioned species. Departure from Hardy-Weinberg expectations was assessed for each microsatellite marker using exact tests (5000 permutations), as implemented in GENEPOP version 3.4 [25]. Linkage disequilibrium was assessed for each microsatellite marker using FSTAT version 2.9.3.2 [26] and with 1000 permutations. The level of significance was adjusted for multiple testing using a sequential Bonferroni correction technique [27].

## Results and Discussion

### Locus identification, validation and polymorphism

Sequencing of the microsatellite-enriched library yielded 18,511 reads. The sequence dataset is available in the Dryad database at http://doi.org/10.5061/dryad.md545. Of these, 5073 (27%) reads contained microsatellite motifs according to QDD analysis. Primer pairs were designed for all loci fulfilling our criteria for primer design [20]. The 146 resulting loci comprised 93 di-, 43 tri-, 5 tetra-,1 penta- and 4 hexanucleotide repeat microsatellites with 5 to 22 repeat units (Table S1). Two *A. vulgare* individuals were genotyped for the 146 microsatellite loci and 41 loci were validated under our amplification conditions and criteria (Table S1). Out of the 41 loci, a first polymorphism analysis based on a 24-individual panel allowed us to identify 33 polymorphic loci (i.e. 80%) (Table S1). Among these 33 polymorphic loci, we selected 14 loci for inclusion in multiplex kits, according to the following criteria: (i) repeat type (larger motifs favored), (ii) number of different alleles scored in 24-individual panel (higher number favored), and (iii) amplification success rate in 24-individual panel (higher number of successfully genotyped individuals favored). The 14 loci were combined in three multiplex kits according to ranges of amplification sizes (Table 1).

We genotyped 40 *A. vulgare* individuals from two field populations with the three multiplex kits. No significant linkage disequilibrium between the different loci was observed after sequential Bonferroni correction. Two loci (AV0023 and AV0061) were monomorphic in the two tested populations (Table 2). However, we kept these two loci in our multiplex kits because they were polymorphic in our initial 24-individual panel, suggesting that they may be informative for other populations. The number of alleles for the 12 other loci ranged from 2 to 6 among the 40 *A. vulgare* individuals, with a mean of 3.9 alleles per locus. Microsatellite loci AV0002, AV0018, AV0056 and AV0099 were highly polymorphic in both populations and they may be particularly relevant markers for analyses requiring high discriminating power, e.g. to investigate paternity in *A. vulgare*. Observed heterozygosity levels varied from 0.1 to 1 in both populations. After sequential Bonferroni correction for multiple testing, loci AV0096 and AV0128 revealed significant excess of heterozygotes in both populations and locus AV0099 showed a significant deficit in La Crèche population. Thus, these loci departed from Hardy-Weinberg equilibrium in concerned populations, likely because of non-exhaustive population sampling. These loci may therefore turn out to be useful for future studies with a more important sampling. The other highlighted polymorphic microsatellites represent a useful set of markers to perform genetic studies on *A. vulgare* and to investigate the impact of *Wolbachia* endosymbionts on *A. vulgare* population genetic structure and evolution.

### Locus transferability in terrestrial isopod species

The 14 newly developed markers were tested in five isopod species. Results are summarized in Table 3. Among these species, *A. nasatum* and *C. elongata* revealed high cross-species transferability with amplification success of 71% (10/14 loci) and 86% (12/14 loci), respectively. Conversely, *O.asellus*, *P. pruinosus* and *P. scaber* revealed moderate amplification success with 21% (3/14 loci), 29% (4/14 loci) and 43% (6/14 loci), respectively. Depending on species, mean number of alleles ranged from 1.8 to 2.8 and mean expected heterozygosity ranged from 0.40 to 0.53. Results from *A. nasatum* are not really surprising given the close phylogenetic relationship with *A. vulgare*. The important rate of successful cross-amplification found in *C.elongata* is more surprising but microsatellite loci show a reduced number of alleles and low heterozygosity indices in *C. elongata* relative to *A. vulgare*. We detected no linkage disequilibrium whereas departure from Hardy-Weinberg expectations was detected for loci AV0056 and AV0096 in *P. scaber* and for loci AV0032 and AV0085 in *A. nasatum*. The deficit in heterozygotes observed for these loci could be explained by the quite small sampling.

## Conclusions

In sum, our work highlights a large set of microsatellite markers useful for studies on *A. vulgare* and other terrestrial isopod species. The polymorphism of these markers now makes it possible to analyze genetic diversity, population structure and reproduction strategies of *A. vulgare* at unprecedented resolution. A study based on these markers is now underway to analyze the impact of *Wolbachia* bacterial endosymbionts on *A. vulgare* nuclear variation. Moreover, these microsatellite markers showed good transferability in five other terrestrial isopod species, in particular in *A. nasatum* and *C. elongata*, for which these microsatellite markers represent promising tools for future genetic studies.

## Supporting Information

Table S1 Information on the 146 microsatellite loci from *Armadillidium vulgare* tested for inclusion in multiplex kits. For each locus the following information is provided: locus name, repeat motif, repeat number, reference sequence, forward primer, reverse primer, PCR product size in reference sequence, and results of experimental tests leading to final selection for three multiplex kits.
(XLSX)
